# Understanding the structural and chemical changes of plant biomass following steam explosion pretreatment

**DOI:** 10.1186/s13068-017-0718-z

**Published:** 2017-02-07

**Authors:** Thomas Auxenfans, David Crônier, Brigitte Chabbert, Gabriel Paës

**Affiliations:** FARE Laboratory, INRA, Université de Reims Champagne-Ardenne, 51100 Reims, France

**Keywords:** Lignocellulose, Biomass, Steam explosion, Pilot scale, Enzymatic saccharification, Biofuels

## Abstract

**Background:**

Biorefining of lignocellulosic biomass has become one of the most valuable alternatives for the production of multi-products such as biofuels. Pretreatment is a prerequisite to increase the enzymatic conversion of the recalcitrant lignocellulose. However, there is still considerable debate regarding the key features of biomass impacting the cellulase accessibility. In this study, we evaluate the structural and chemical features of three different representative biomasses (*Miscanthus* × *giganteus*, poplar and wheat straw), before and after steam explosion pretreatment at increasing severities, by monitoring chemical analysis, SEM, FTIR and 2D NMR.

**Results:**

Regardless the biomass type, combined steam explosion pretreatment with dilute sulfuric acid impregnation resulted in significant improvement of the cellulose conversion. Chemical analyses revealed that the pretreatment selectively degraded the hemicellulosic fraction and associated cross-linking ferulic acids. As a result, the pretreated residues contained mostly cellulosic glucose and lignin. In addition, the pretreatment directly affected the cellulose crystallinity but these variations were dependent upon the biomass type. Important chemical modifications also occurred in lignin since the β-*O*-4′ aryl-ether linkages were found to be homolytically cleaved, followed by some recoupling/recondensation to β-β′ and β-5′ linkages, regardless the biomass type. Finally, 2D NMR analysis of the whole biomass showed that the pretreatment preferentially degraded the syringyl-type lignin fractions in miscanthus and wheat straw while it was not affected in the pretreated poplar samples.

**Conclusions:**

Our findings provide an enhanced understanding of parameters impacting biomass recalcitrance, which can be easily generalized to both woody and non-woody biomass species. Results indeed suggest that the hemicellulose removal accompanied by the significant reduction in the cross-linking phenolic acids and the redistribution of lignin are strongly correlated with the enzymatic saccharification, by loosening the cell wall structure thus allowing easier cellulase accessibility. By contrast, we have shown that the changes in the syringyl/guaiacyl ratio and the cellulose crystallinity do not seem to be relevant factors in assessing the enzymatic digestibility. Some biomass type-dependent and easily measurable FTIR factors are highly correlated to saccharification.

## Background

Fermentescible sugars derived from lignocellulosic biomasses (LCBs), such as dedicated crops, agricultural and wood residues, have considerable potential for the production of biofuels (i.e. cellulosic ethanol), chemicals and biomaterials [1]. LCBs indeed contain high amounts of carbohydrates (i.e. cellulose and hemicelluloses) but the complex and heterogeneous cell wall architecture makes them recalcitrant to cellulase-catalysed hydrolysis and subsequent ethanolic fermentation [2]. This biomass recalcitrance is closely related to chemical and physical features, such as the highly ordered structure of cellulose fibrils which are embedded in a complex matrix of cross-linked hemicellulose, pectins, glycosylated proteins and lignin as well as the degree of lignification [3, 4]. The presence of lignin and its cross-linking with other cell wall constituents (lignin carbohydrates complexes, LCCs) is indeed considered as one of the most significant contributors to LCB recalcitrance [5–9]. The composition of lignin, its physical distribution, as well as the ratio of syringyl (*S*) unit to guaiacyl (*G*) unit also have a significant negative impact on the cellulase-catalysed hydrolysis since lignin acts as both a physical barrier, restricting the cellulose accessibility, and as a cellulase non-productive binder [7, 10–13].

Pretreatment step is thus required to overcome LCB recalcitrance and make polysaccharides easily enzymatically digestible. To date, several pretreatment technologies have been developed which can broadly be categorized into biological, physical, chemical, physico-chemical methods and by some combinations of these processes [14]. Generally, the pretreatment step aims to reduce the lignin and/or the hemicellulose content, simultaneously disrupting the plant cell wall architecture thus increasing surface area and cellulose accessibility [15]. Among these pretreatments, steam explosion has been increasingly considered as one of the most efficient, environmentally friendly and cost-effective processes for industrial application and, thus, has been widely tested at the pilot scale for various LCBs [16–18]. Steam explosion involves exposing LCB to high-pressure saturated steam and then reducing pressure swiftly, making the materials undergoing an explosive decompression. This results in the breakdown of the lignocellulosic matrix, partial removal and/or redistribution of lignin and significant enhancement of the cellulose accessibility [16]. A presoaking step using dilute acid that efficiently solubilizes hemicelluloses can be added before steam explosion. Linkages between hemicellulose and cellulose are broken, improving the subsequent steam explosion efficiency. Hence, the dilute acid presoaked steam explosion process is considered to be close to industrial application [19].

Several studies investigating the structural changes of lignin in steam exploded LCBs have been reported using different methods such as nuclear magnetic resonance (NMR), size exclusion chromatography (SEC) and nitrobenzene oxidation [20–23]. Some limitations nonetheless remain for the investigation of lignin transformations since milled wood lignin (MWL), alkali-extracted lignin, enzymatic mild acidolysis lignin and cellulolytic enzyme lignin (CEL) are often selected as references. Indeed, this methodology would not seem to be the most appropriate because these isolated lignins (i) might have their structure modified according to the isolation method [24] and (ii) are isolated from preferential locations in the plant cell wall, such as middle lamella for MWL (more guaiacyl-type units) and secondary wall S_2_ for CEL (mainly syringyl-type units). Based on these limitations, there is a need to elucidate the chemical lignin transformations involved during the pretreatment without competing reactions due to the isolation method.

The aim of this work is to investigate in greater details the key structural features of steam exploded samples varying both in LCB type and in pretreatment severity. Three distinct representative LCBs for second generation bioethanol production were selected as follows: dedicated crops (*Miscanthus* × *giganteus*); agricultural residues (wheat straw) and short rotation coppices (poplar). Steam explosion with dilute sulfuric acid presoaking was the selected pretreatment. Then, structural changes of the steam exploded samples were investigated by a series of complementary spectral and chemical approaches such as two-dimensional heteronuclear single-quantum coherence (2D HSQC) NMR, Fourier transform infrared (FTIR) spectroscopy and scanning electron microscopy (SEM). Overall, analysis of data obtained highlight the features correlated to LCB digestibility.

## Results and discussion

**Fig. 1:**
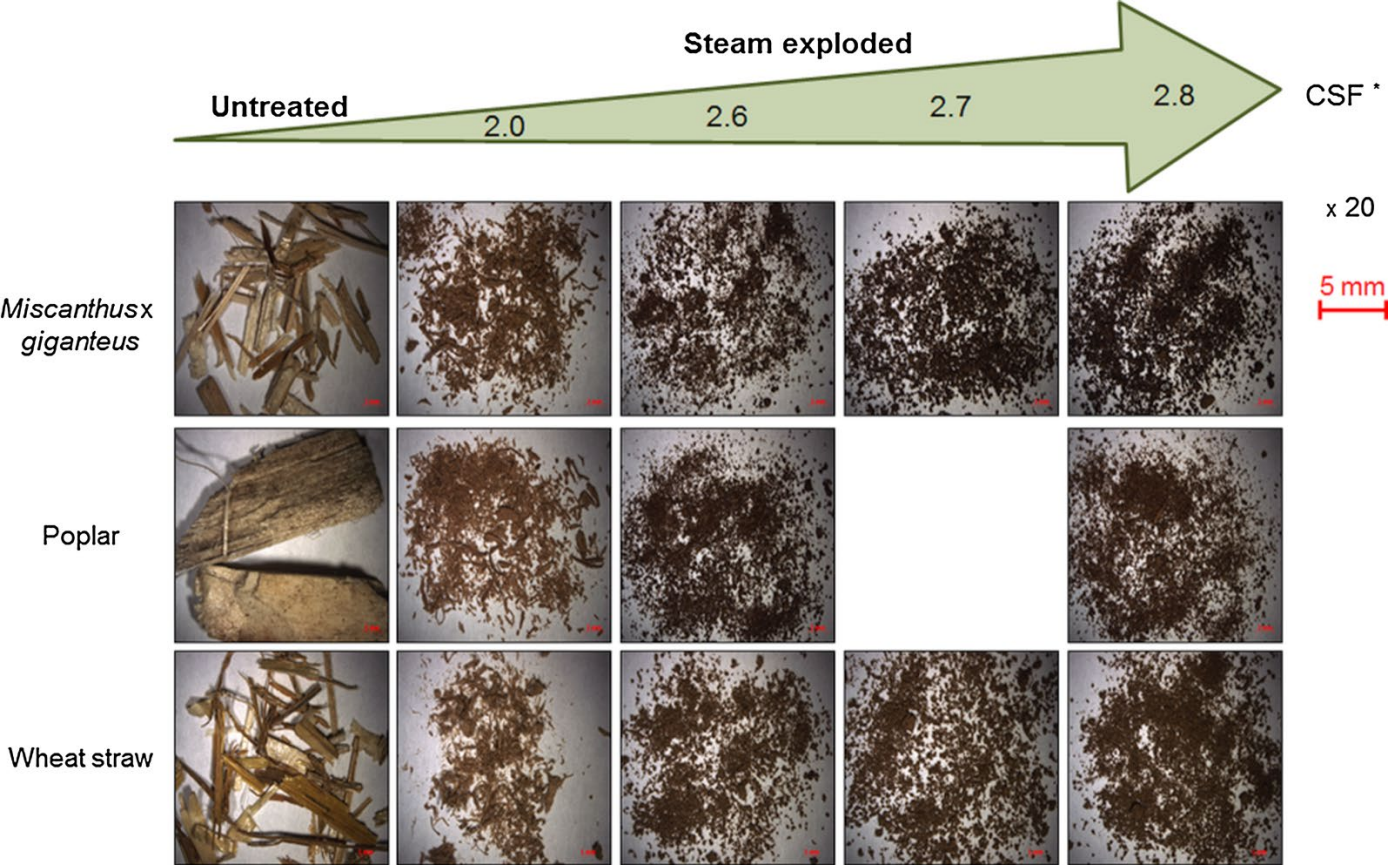
Morphological changes in LCBs after steam explosion pretreatment with increasing severities (binocular magnification × 20). *CSF combined severity factor

Figure 1 shows the set of 14 samples obtained for the different LCB types (*Miscanthus* × *giganteus*, poplar and wheat straw). The combined severity factor (CSF), commonly used for the comparison of steam explosion treatments at different temperatures and times, was calculated for each experiment. Low-magnification imaging of the pretreated samples shows disintegration of LCB cell wall structure into finer components compared to the untreated material. As a result, the higher the pretreatment severity is, the smaller mean particle size gets. The particle colour also becomes more brownish with increasing CSF, revealing the relatively large presence of residual lignin.

### Enzymatic saccharification

**Fig. 2:**
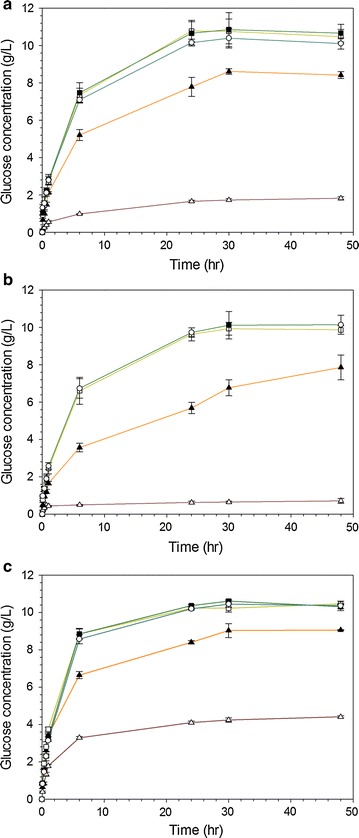
Kinetics curves of glucose production during cellulase-catalysed hydrolysis of **a** *Miscanthus* × *giganteus*; **b** poplar and **c** wheat straw residues. *Triangle* Untreated substrates and pretreated with increasing pretreatment CSF: *filled triangle* 2.0; *square* 2.6; *filled square* 2.7 and *circle* 2.8

Enzymatic saccharification of both native and pretreated LCB samples was performed using commercial cellulase preparation Cellic CTec2® at a protein loading of 40 FPU/g-glucan. Figure 2 presents the profile of glucose release during a 48 h-cellulase-catalysed hydrolysis of *Miscanthus* × *giganteus* (Fig. 2a), poplar (Fig. 2b) and wheat straw (Fig. 2c). Both the glucose concentration released and the initial apparent rates of glucose are also reported in Table 1.

**Table 1:** Glucose concentration at 24 and 48 h and initial apparent rates of glucose production during enzymatic saccharification of lignocellulosic substrates

Substrate	Pretreatment severity (CSF)	Glucose concentration (g/L)^a^		Initial apparent rate (mg/L/min)
		24 h	48 h	
*Miscanthus* × *giganteus*	Untreated	1.66 ± 0.06	1.82 ± 0.09	10.8 ± 1.4
	2.0	7.79 ± 0.51	8.42 ± 0.18	46.7 ± 3.3
	2.6	10.78 ± 0.57	10.48 ± 0.38	68.7 ± 4.4
	2.7	10.67 ± 0.62	10.67 ± 0.47	71.2 ± 3.4
	2.8	10.16 ± 0.18	10.11 ± 0.30	70.8 ± 3.3
Poplar	Untreated	0.62 ± 0.07	0.71 ± 0.13	20.1 ± 1.9
	2.0	5.68 ± 0.30	7.87 ± 0.66	42.5 ± 5.2
	2.6	9.64 ± 0.35	9.87 ± 0.23	56.6 ± 3.1
	2.8	9.73 ± 0.25	10.15 ± 0.51	58.1 ± 7.5
Wheat straw	Untreated	4.10 ± 0.08	4.41 ± 0.06	41.9 ± 5.0
	2.0	8.40 ± 0.10	9.06 ± 0.02	82.2 ± 8.9
	2.6	10.23 ± 0.09	10.46 ± 0.02	91.7 ± 11.4
	2.7	10.37 ± 0.06	10.31 ± 0.10	67.1 ± 6.7
	2.8	10.20 ± 0.04	10.36 ± 0.25	68.1 ± 1.9

^a^Expressed as mean ± SD of triplicate

The enzymatic saccharification profiles present similar patterns, regardless of LCB type, as shown in Fig. 2. The cellulose conversion is indeed highly improved as the different substrates undergo the steam explosion with dilute acid presoaking pretreatment, without considering its severity (Fig. 2). While only a small fraction of the cellulose was converted into glucose in the untreated miscanthus and poplar substrates at 48 h, a 5- to 15-fold increase in released glucose was achieved in the pretreated samples, as outlined in Table 1. Furthermore, an increase in the steam explosion severity from 2.0 to 2.8 resulted in an increase in glucose release of 20% at 48 h (*p* = 0.001) (Fig. 2a, b). These observations are further supported by the significant improvement (*p* < 0.001) of the initial apparent rates of glucose production (Table 1). Similarly, while increasing the pretreatment severity for wheat straw, the enzymatic saccharification was also much faster (Table 1) but the concentrations of glucose released were statistically similar (*p* = 0.072) at 48 h (Fig. 2c). Interestingly, although initial apparent hydrolysis rates were largely improved after pretreatment, the highest severities led to a slight reduction of them (*p* = 0.041), probably due to the presence of higher inhibitors amounts of cellulase in the hydrolysis medium, such as free xylan mono/oligomers [25].

By contrast, the increase in the pretreatment CSF from 2.6 to 2.8 did not appear to significantly increase the cellulase-catalysed hydrolysis efficiency, since both the initial apparent rates of glucose production and concentrations at 48 h achieved for each type of presoaked LCB were not statistically different (Fig. 2; Table 1). From these results, the steam explosion pretreatment combined with dilute sulfuric acid impregnation constitutes a versatile pretreatment of various LCBs including woody (poplar) and non-woody (miscanthus and wheat straw) species. In addition, in agreement with literature data, these relative mild processes conditions were found to be more favourable preventing both thermal degradation of cellulose into sugars and formation of inhibitory compounds [26].

### Morphological properties

**Fig. 3:**
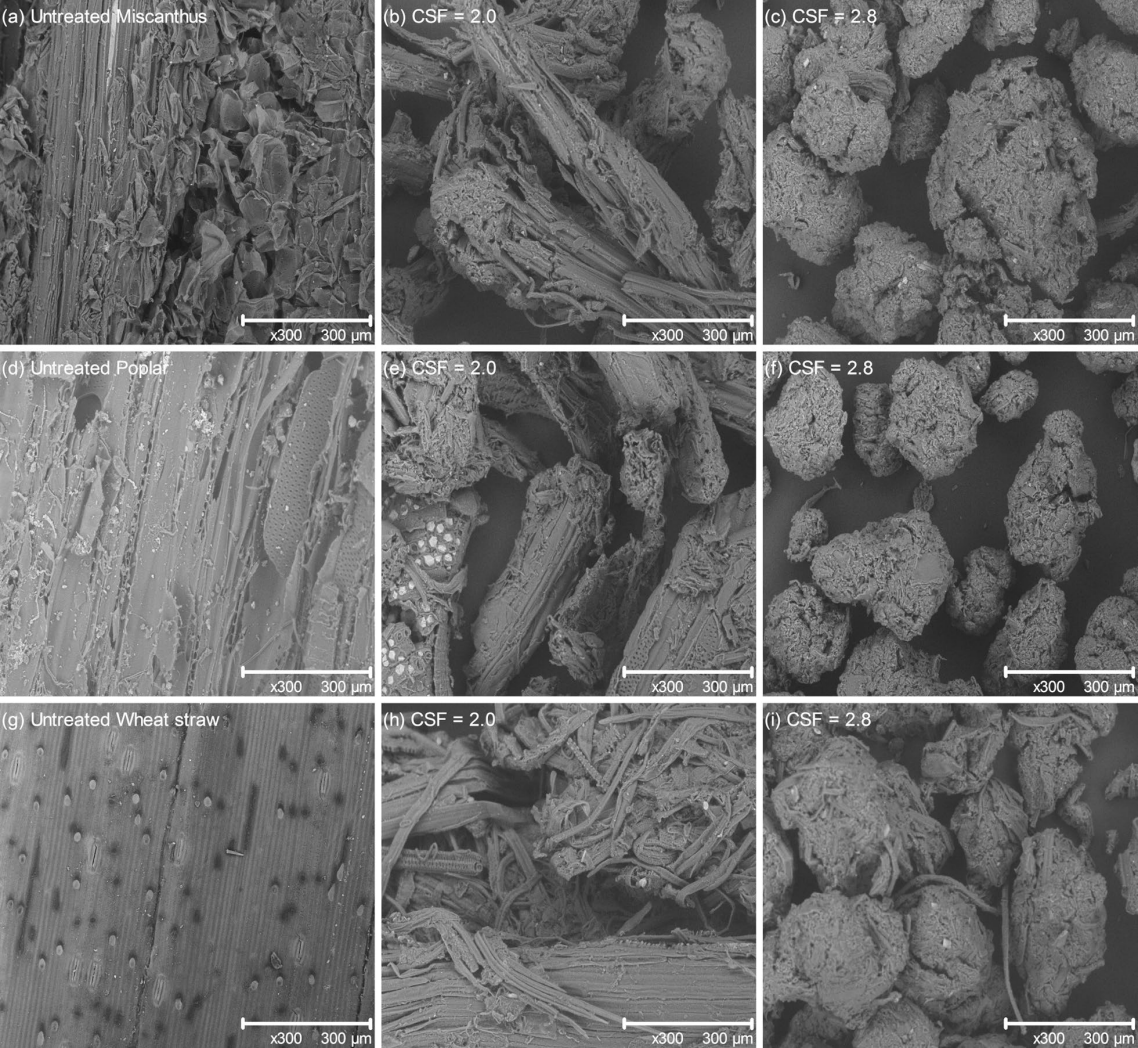
SEM micrographs (×300) of untreated and pretreated **a**–**c** miscanthus; **d**–**f** poplar and **g**–**i** wheat straw samples with increasing pretreatment severities

To better understand the significant enhancement of LCB enzymatic digestibility after pretreatment, the morphology of the untreated and pretreated samples was investigated by SEM, as presented in Fig. 3. Native miscanthus (Fig. 3a), poplar (Fig. 3d) and wheat straw (Fig. 3g) samples exhibited rigid, compact fibrillary morphology with thick-walled fibre cells and fibres constituted by parallel stripes, limiting the cellulose accessibility. By contrast, steam exploded pretreated samples presented a more disorganized morphology characterized by the separation and greater exposure of fibres as well as loosening of the fibrous network, which may be due to the solubilisation of cell wall components. The most exposed cell wall structure likely allows greater cellulose accessibility to cellulase. This impact is directly correlated to the pretreatment severity since some persistent cell wall structures are still present after pretreatment in moderate reactive conditions (Fig. 3b, e, h) while the pretreated samples at high severity (Fig. 3c, f, i) appear as large aggregates of fibrous tissue with cylindrical shape.

### Chemical composition

The chemical composition of both untreated and pretreated samples was then investigated, as presented in Table 2. Cellulose, hemicelluloses, lignin and ash contents were determined on a dry weight basis. Hemicelluloses comprise xylose, arabinose, galactose, rhamnose, mannose and uronic acids, while the total lignin amount was expressed as the sum of the acid soluble and insoluble lignin concentrations. For the raw materials (*Miscanthus* × *giganteus*, poplar and wheat straw), the contents in cellulose (i.e. glucose), hemicelluloses and lignin were in the ranges 39.1–45.3, 20.2–29.4 and 24.6–29.5% (w/w), respectively (Table 2). After pretreatment and regardless its severity, the chemical composition of the pretreated residues was in the same order (Table 2). Pretreated LCBs indeed exhibited an effective relative enrichment (*p* < 0.001) in cellulose and lignin, mainly caused by the hemicellulosic fraction removal. Worth noting is that this impact is higher as the pretreatment severity increases (*p* < 0.005). As indicated in Table 2, about 90% of the hemicellulose fraction was removed from the pretreated substrate under the most severe pretreatment conditions (CSF = 2.8), regardless the LCB. Therefore, steam explosion pretreatment performs an effective breakdown of LCB components, mainly achieved by the hemicellulosic fraction removal, thus improving the cellulose accessibility to enzymes in the subsequent enzymatic hydrolysis step.

**Table 2:** Chemical composition of untreated and steam exploded lignocellulosic biomasses

Lignocellulosic biomass	Pretreatment severity (CSF)	Moisture content	Glucose	Hemicellulose^a^	AIL^b^	ASL^c^	Total lignin^d^	Ash
*Miscanthus* × *giganteus*	Untreated	3.3 ± 0.2	45.3 ± 0.8	25.3 ± 0.7	23.8 ± 0.5	1.7 ± 0.1	25.5 ± 0.6	0.5 ± 0.1
	2.0	2.8 ± 0.1	65.5 ± 3.4	5.6 ± 0.4	25.7 ± 0.5	1.5 ± 0.2	27.2 ± 0.7	0.5 ± 0.1
	2.6	2.5 ± 0.3	66.7 ± 2.7	1.9 ± 0.5	27.3 ± 0.4	1.8 ± 0.1	29.1 ± 0.5	0.7 ± 0.4
	2.7	2.8 ± 0.2	65.8 ± 1.9	2.0 ± 0.1	27.8 ± 0.9	1.8 ± 0.1	29.6 ± 1.0	0.8 ± 0.3
	2.8	3.3 ± 0.1	64.7 ± 4.1	1.8 ± 0.3	27.7 ± 0.3	2.0 ± 0.1	29.7 ± 0.4	0.9 ± 0.1
Poplar	Untreated	3.9 ± 0.3	43.2 ± 2.6	20.2 ± 1.3	27.8 ± 0.2	1.7 ± 0.1	29.5 ± 0.3	0.5 ± 0.2
	2.0	3.0 ± 0.1	59.4 ± 3.3	6.4 ± 1.5	28.8 ± 0.1	1.7 ± 0.1	30.5 ± 0.2	0.4 ± 0.1
	2.6	3.2 ± 0.1	61.6 ± 3.3	2.0 ± 0.4	31.0 ± 0.5	2.0 ± 0.1	33.0 ± 0.6	0.8 ± 0.1
	2.8	3.2 ± 0.1	62.7 ± 1.1	1.6 ± 0.2	31.1 ± 0.6	2.3 ± 0.1	33.4 ± 0.7	0.8 ± 0.1
Wheat straw	Untreated	5.2 ± 0.1	39.1 ± 2.2	29.4 ± 1.8	22.6 ± 0.5	2.0 ± 0.1	24.6 ± 0.6	2.0 ± 0.3
	2.0	3.6 ± 0.1	59.5 ± 1.6	10.0 ± 0.4	23.0 ± 0.3	1.6 ± 0.1	24.6 ± 0.4	1.3 ± 0.2
	2.6	3.4 ± 0.3	63.4 ± 2.0	4.3 ± 0.3	25.4 ± 0.4	1.9 ± 0.1	27.3 ± 0.5	1.5 ± 0.3
	2.7	3.1 ± 0.1	63.7 ± 2.3	3.9 ± 0.2	25.2 ± 0.4	2.0 ± 0.1	27.2 ± 0.5	1.7 ± 0.3
	2.8	3.3 ± 0.1	63.2 ± 0.7	3.4 ± 0.1	28.0 ± 0.3	2.1 ± 0.1	30.1 ± 0.4	2.0 ± 0.1

Compositions are expressed as percentage (% w/w) of the residues

^a^Hemicellulose (%) = Xyl (%) + Ara (%) + Gal (%) + Rha (%) + Man (%) + galacturonic acid (%) + glucuronic acid (%)

^b^Acid insoluble Klason lignin

^c^Acid soluble lignin

^d^Total lignin (%) = acid insoluble lignin (%) + acid soluble lignin (%)

Another important point is that lignin contents were in the same range for both the untreated and pretreated LCBs (Table 2). Therefore, the higher susceptibility to cellulase-catalysed hydrolysis observed for the pretreated LCBs cannot be explained solely through the lignin content and other factors related to the physico-chemical characteristics of the LCB samples also play a role.

### FTIR characterization

**Fig. 4:**
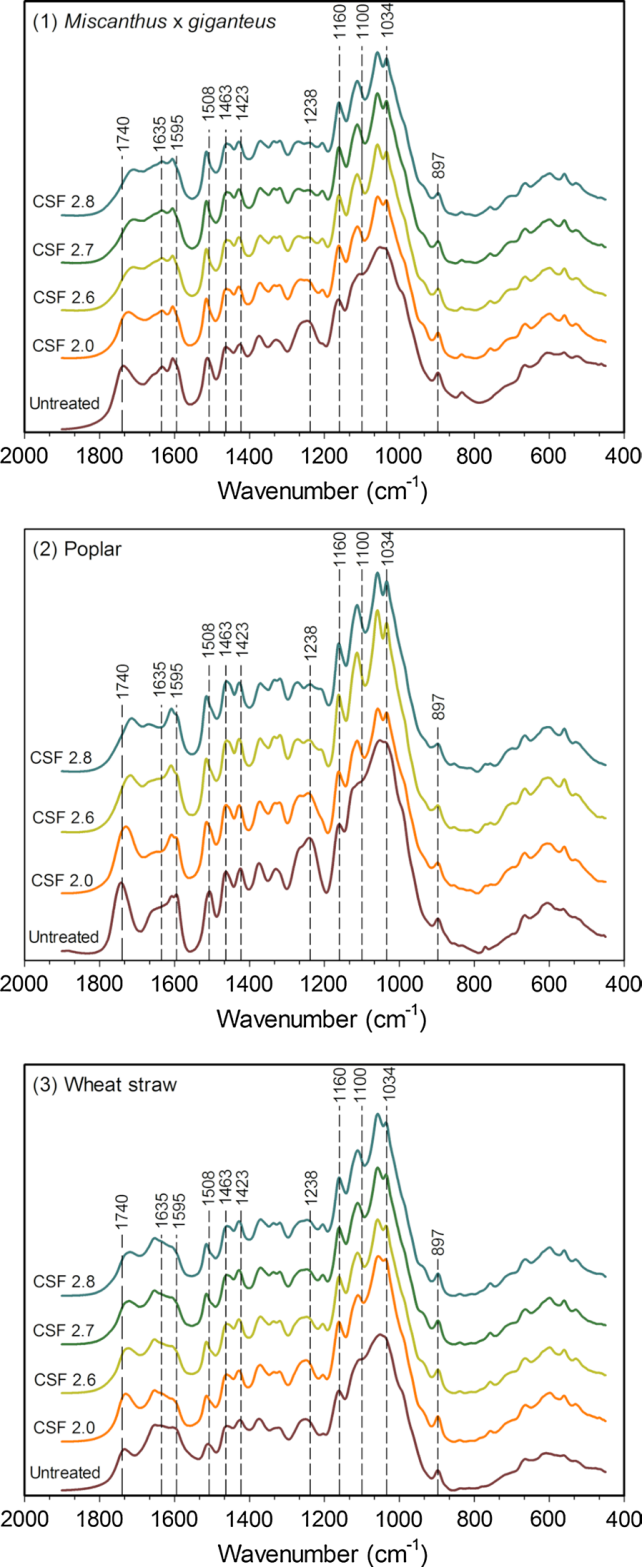
FTIR spectra of untreated and steam exploded samples with increasing pretreatment severities

To investigate the chemical modifications after pretreatment, an analysis of the LCB samples was undertaken using FTIR spectroscopy. Regardless the LCB, the peaks in the 890–1200 cm^-1^ region associated with the carbohydrate abundance became more distinct (Fig. 4). Indeed, the bands at 1034, 1100 and 1160 cm^-1^ correspond to the C–O/C–H stretching of sugars in the cellulose and C–H stretching of sugars and C–O–C stretching of β-(1,4) glycosidic bond between sugars [27]. One important feature likely explaining the enhancement of the enzymatic digestibility generally involves the changes in cellulose structure. Using the FTIR data, several peak ratios have earlier been described highlighting the differences between samples with regard to the relative amorphous and crystalline cellulose amounts [28, 29]. An empirical crystallinity index, also known as lateral order index (LOI), was defined as the ratio between intensity of the bands at 897 cm^-1^ (glycosidic bond β-(1,4) in cellulose) and 1420–1430 cm^-1^ which is associated with the amount of the crystalline structure of cellulose [29]. In addition, the hydrogen bond intensity (HBI) is closely related to the crystal system and the degree of intermolecular regularity, i.e. crystallinity as well as the amount of bound water [30, 31]. This parameter is determined from the ratio of intensities at 3400 cm^-1^ (O–H stretching, H-bonds between molecules) and 1320 cm^-1^ (CH rocking vibration of the glucose ring).

For both *Miscanthus* × *giganteus* and wheat straw samples, the LOI showed higher value (*p* < 0.001) for the pretreated samples compared to the untreated material (Table 3), suggesting a more ordered cellulose structure after pretreatment [32]. Surprisingly, pretreated poplar showed opposite effect since LOI values significantly decreased (*p* < 0.001) after pretreatment (Table 3) [30, 31]. In the same way, HBI of pretreated miscanthus and wheat straw samples is significantly higher than untreated ones (*p* < 0.001) while the values for poplar were slightly decreased after pretreatment (*p* = 0.040), thus agreeing with the changes in LOI values. These results indicate that the pretreated miscanthus and wheat straw samples contain much more cellulose chains in a highly organized form than the untreated ones. This results in higher hydrogen bond intensity between neighbour cellulose chains and in more packed cellulose structure as well as higher crystallinity. This could be attributed to the facts that the pretreatment at high temperature led to a reorganization of the amorphous and paracrystalline cellulose regions in these LCBs [32]. As for LOI, an opposite trend is observed for the pretreated poplar samples when increasing the pretreatment severity. A possible explanation might originate from the structural difference between the woody (poplar) and grass (*Miscanthus* × *giganteus* and wheat straw) LCBs. Overall, these results demonstrate that the features related to the cellulose structure and especially its crystallinity are not the most reliable factors to explain the higher enzymatic digestibility of the pretreated samples.

**Table 3:** Cellulose- and lignin-related properties of native and pretreated biomass

Substrate	Pretreatment severity (CSF)	LOI	HBI	CLL
		*A*_1423_/*A*_897_	*A*_3400_/*A*_1323_	*A*_1508_/*A*_1600_
*Miscanthus* × *giganteus*	Untreated	0.74 ± 0.03	15.5 ± 0.7	1.64 ± 0.03
	2.0	0.79 ± 0.01	16.7 ± 0.3	2.01 ± 0.02
	2.6	0.94 ± 0.03	18.0 ± 0.3	2.51 ± 0.08
	2.7	0.89 ± 0.03	18.6 ± 0.3	2.48 ± 0.07
	2.8	0.94 ± 0.04	18.6 ± 0.7	2.38 ± 0.09
Poplar	Untreated	1.77 ± 0.04	11.9 ± 0.1	1.32 ± 0.04
	2.0	1.43 ± 0.04	10.7 ± 0.2	1.28 ± 0.01
	2.6	1.44 ± 0.02	11.1 ± 0.3	1.38 ± 0.03
	2.8	1.44 ± 0.06	11.1 ± 0.4	1.35 ± 0.01
Wheat straw	Untreated	0.78 ± 0.03	20.9 ± 0.7	0.91 ± 0.03
	2.0	0.80 ± 0.03	25.5 ± 0.6	2.35 ± 0.52
	2.6	0.83 ± 0.03	23.7 ± 0.4	2.94 ± 0.23
	2.7	0.87 ± 0.02	24.6 ± 0.4	2.23 ± 0.19
	2.8	0.90 ± 0.01	23.5 ± 0.6	2.28 ± 0.09

*LOI* lateral order index, *HBI* hydrogen bond intensity, *CLL* cross-linked lignin

All the values are expressed as mean ± SD of triplicate

Increased severity was also associated with a disappearance or a significant decrease in the intensity of peaks at 1740; 1635 and 1238 cm^-1^ (Fig. 4), which are related to C–O stretching, O–H bending and the stretching bands C–O and C–H in the hemicelluloses, respectively, confirming the chemical composition analyses (Table 2) [33]. More interestingly, the region between 1300 and 1600 cm^-1^ is a large contribution of the aromatic skeletal vibrations of lignin. The bands at 1600, 1508 and 1463 cm^-1^ are reported to be stretching of the C=C and C=O aromatic lignin; the deformation of lignin CH_2_ and CH_3_ and the C=C stretching of the aromatic ring in lignin, respectively [27]. As the pretreatment severity increased, the intensity of the band at 1600 cm^-1^ thus decreased while the band at 1508 and 1463 cm^-1^ increased in the pretreated miscanthus and wheat straw samples (Fig. 4). Using the ratio between band intensities at 1600 and 1508 cm^-1^, it is possible to evaluate the proportion of lignin with condensed and cross-linked structures which is a characteristic feature of the concentration in guaiacyl, known as the cross-linked lignin ratio (CLL) [34, 35]. As presented in Table 3, the higher the pretreatment severity is, the higher the CLL value is for miscanthus and wheat straw samples (*p* < 0.001), reflecting an enrichment in lignin with condensed and cross-linked G-type lignin structures. One explanation could be that lignin is solubilized during the pretreatment and then repolymerized/recondensed. By contrast, CLL values of the pretreated poplar samples were not statistically different compared to the untreated ones (*p* = 0.217), confirming that the impact of the pretreatment strongly depends on the LCB type. FTIR results concur with the chemical analyses (Table 2) and also suggest that the increment of the pretreatment severity could enhance the extent of structural modifications of lignin.

### Effect of pretreatment on phenolic monomers

**Fig. 5:**
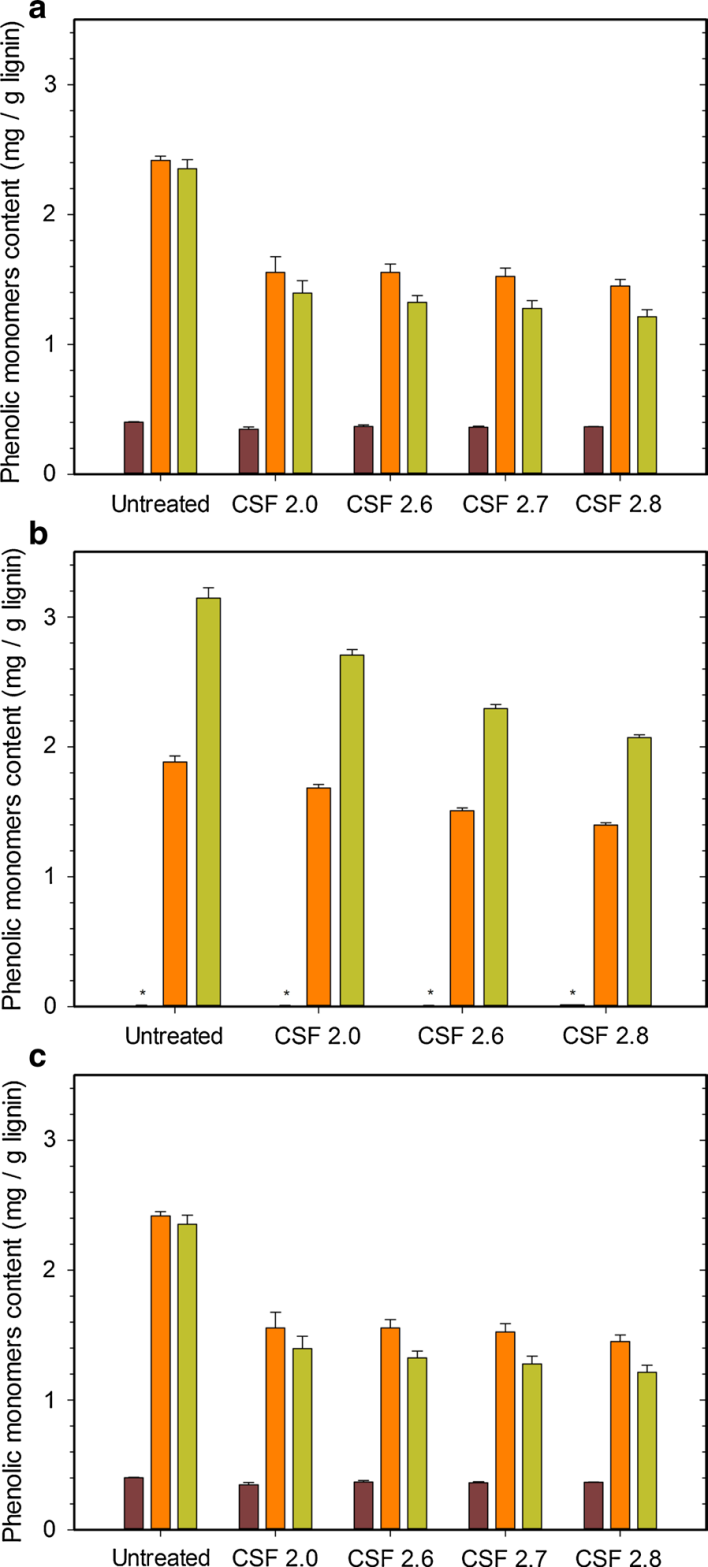
Phenolic monomers composition (mg/g lignin) of **a** *Miscanthus* × *giganteus*; **b** poplar and **c** wheat straw samples without pretreatment and pretreated with increasing severity: *red* 4-hydroxybenzoic acid; *orange* vanillyl-type monomers and *green* syringyl-type monomers. * Not detectable

The quantification of the total phenolic monomers was performed using alkaline hydrolysis at 170 °C. The composition and quantity of the aromatic acids and aldehydes reflect the structural features of the aromatic ring and the extent of uncondensed linkages between lignin monomers [36, 37]. Vanillyl-type monomers comprising vanillin and vanillic acid while syringyl-type monomers were determined as the sum of the concentrations of syringic acid and syringaldehyde. It is worth to notice that vanillin and vanillic acid are produced by degradation of guaiacyl units of lignin; both syringaldehyde and syringic acid derived from the degradation of syringyl units of lignin and 4-hydroxybenzoic acid arises from *para*-hydroxyphenyl (H) units of lignin [36]. As illustrated in Fig. 5, the phenolic profiles in vanillyl-type, syringyl-type and 4-hydroxybenzoic acid monomers obviously differ significantly between the three untreated LCBs, reflecting the differences in lignin structure and content between them. The lignin in poplar indeed mostly comprises syringyl units, together with smaller proportions of guaiacyl units while grass species, such as miscanthus and wheat straw are generally composed of guaiacyl and syringyl units in roughly equal amounts as well as small quantities of *para*-hydroxyphenyl units [38–41]. After pretreatment and regardless the LCB, the composition in vanillyl-type, syringyl-type and 4-hydroxybenzoic acid monomers follow the same trend (Fig. 5). As the pretreatment severity increases and whatever the LCB is, the pretreated residues exhibited a significant decrease (*p* < 0.001) in both vanillyl- and syringyl-type monomers whereas the 4-hydroxybenzoic acid concentration in pretreated miscanthus and wheat straw did not statistically differ. Under the most severe pretreatment conditions, the respective concentration of syringyl- and vanillyl-type monomers was thus about 1.4–2 times and 1.3–1.7 time lower in the pretreated residues, respectively. Nevertheless, only a few percent of the aromatic monomers were detected in the pretreated LCBs although samples contained about 30% (w/w) of lignin (Table 2). The content of aryl-ether (β-*O*-4′) linkages (uncondensed bound) which are disrupted by the alkali hydrolysis used for phenolic quantification has thus decreased after pretreatment. These results are likely attributed to the condensation reactions during pretreatment, resulting in a lignin structure which is much less easily degraded into phenolic monomers.

Both ester- and ether-linked phenolic acids were also determined. Ester- and ether-linked ferulic acid (FA) and *p*-coumaric acid (*p*-CA) are the major hydroxycinnamates bound to grass cell walls in contrast to woody species [38, 39, 42–44]. Accordingly, untreated and treated poplar exhibited much smaller quantities than the other two LCBs (data not shown). The amounts of *p*-CA and FA were quantified for miscanthus and wheat straw (Table 4) and expressed in the relation with the total lignin and hemicellulose content, respectively (Table 2) [43, 44]. FA is mostly ester-linked to hemicelluloses or ether-linked to lignin to form LCCs via ferulic bridges, contributing to the cell wall cross-linking [38, 42–44]. The concentrations of both ester- and ether-linked FA were in the same range in these two LCB and were drastically decreased as the pretreatment severity increased until more than 90% of the total FA fraction was removed (Table 4). This loss is strongly correlated with the significant decrease of the hemicellulose fraction in the pretreated miscanthus and wheat straw samples, showing that the pretreatment caused an auto-catalysed cleavage of the glycosidic linkages in hemicelluloses as well as the ester and ether bonds involved in the LCCs. The reduction in the inter-polymeric cross-linking such as those mediated via FA would thus be expected to facilitate the extraction of hemicellulose, especially arabinoxylans and consequently reducing the steric hindrance and enhancing the cellulase accessibility.

**Table 4:** Ether- and Ester-linked *p*-coumaric and ferulic acid content in native and steam exploded biomasses

Substrate	Pretreatment severity (CSF)	*p*-coumaric acid (mg/g lignin)			Ferulic acid (mg/g hemicellulose)		
		Ester-linked^a^	Ether-linked^b^	Total^c^	Ester-linked^a^	Ether-linked^b^	Total^c^
*Miscanthus* × *giganteus*	Untreated	3.61 ± 0.11	1.20 ± 0.11	4.81 ± 0.08	1.20 ± 0.04	0.82 ± 0.04	2.02 ± 0.03
	2.0	3.18 ± 0.02	0.33 ± 0.05	3.50 ± 0.05	0.21 ± 0.01	0.14 ± 0.01	0.35 ± 0.01
	2.6	2.84 ± 0.08	0.34 ± 0.08	3.18 ± 0.04	0.04 ± 0.00	0.06 ± 0.00	0.10 ± 0.01
	2.7	2.63 ± 0.04	0.39 ± 0.12	3.03 ± 0.08	0.05 ± 0.00	0.05 ± 0.00	0.10 ± 0.01
	2.8	2.66 ± 0.06	0.30 ± 0.07	2.96 ± 0.04	0.05 ± 0.00	0.04 ± 0.00	0.09 ± 0.01
Wheat straw	Untreated	0.82 ± 0.01	0.33 ± 0.01	1.15 ± 0.01	1.22 ± 0.01	0.67 ± 0.01	1.89 ± 0.02
	2.0	0.97 ± 0.01	0.20 ± 0.03	1.18 ± 0.01	0.35 ± 0.03	0.26 ± 0.04	0.60 ± 0.01
	2.6	0.97 ± 0.03	0.24 ± 0.02	1.21 ± 0.01	0.09 ± 0.01	0.16 ± 0.01	0.26 ± 0.03
	2.7	0.96 ± 0.01	0.22 ± 0.01	1.17 ± 0.01	0.08 ± 0.01	0.14 ± 0.01	0.23 ± 0.01
	2.8	0.95 ± 0.02	0.18 ± 0.02	1.14 ± 0.01	0.08 ± 0.01	0.11 ± 0.01	0.19 ± 0.04

All the values are expressed as mean ± SD of triplicate

^a^Represents the ester-linked fraction released during 2 M NaOH hydrolysis at 35 °C for 2 h

^b^Represents the difference between the ester-linked fraction and the total fraction released during NaOH hydrolysis

^c^Represents the ester- and the ether-linked fraction released during 4 M NaOH hydrolysis at 170 °C for 2 h

By contrast, *p*-CA is known to be mainly esterified with lignin, especially with the syringyl moieties at the γ-position of the side chain [42, 45]. The data in Table 4 revealed that miscanthus contained larger amount of *p*-CA than wheat straw as previously shown [46, 47]. The content of *p*-CA decreased from 4.81 to 2.96 mg/g lignin in the pretreated miscanthus samples with increased pretreatment severity. On the other hand, the content in *p*-CA remained around 1.17 mg/g lignin in the pretreated wheat straw whatever the pretreatment severity was (Table 4). More importantly, the higher values of *p*-CA than those of FA in the pretreated residues indicated that substantial amounts of *p*-coumaric acids remained tightly esterified and etherified to the lignin and were not cleaved during the pretreatment. Contrarily, nearly all FAs which are ester-linked to hemicelluloses could be easily removed. These results are consistent with our assumption that the pretreatment would mainly lead to modifications and reorganization in lignin structure rather than to its removal.

### 2D HSQC NMR characterization

**Fig. 6:**
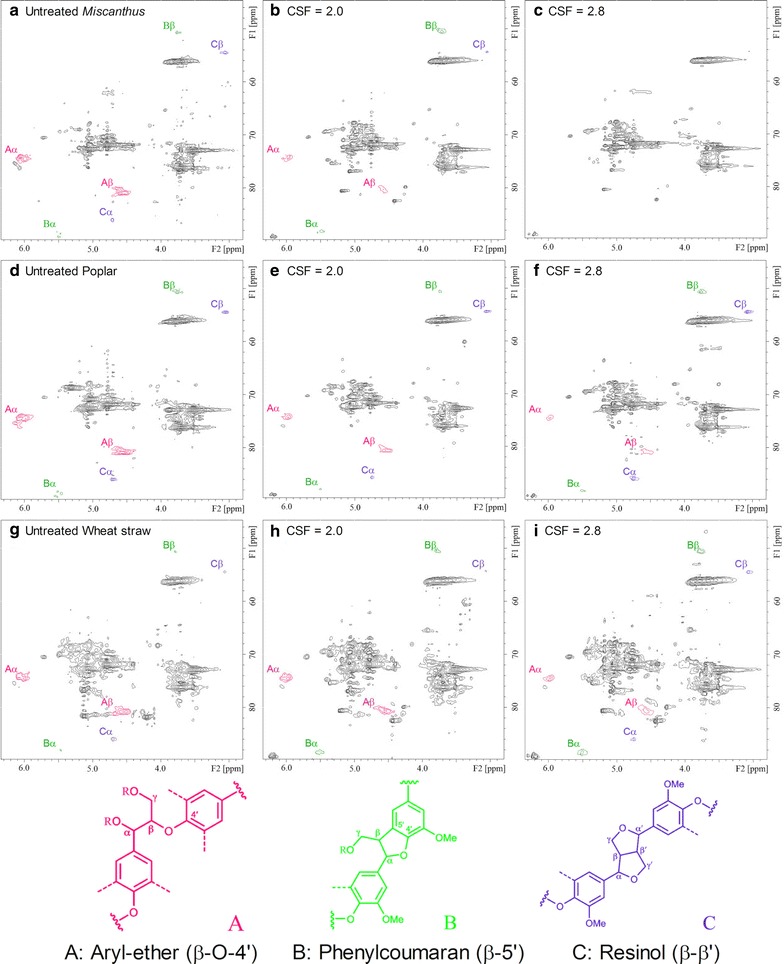
Side-chain in the 2D HSQC NMR spectra of the untreated and pretreated **a**–**c** *Miscanthus* × *giganteus*; **d**–**f** poplar and **g**–**i** wheat straw samples

To obtain further precise information on the effect of pretreatment severity on the lignin structural features, both untreated and pretreated acetylated samples were investigated by 2D HSQC NMR, which allows characterization of the whole lignin in contrast to wet chemical analysis. NMR spectra are given in Fig. 6 and peaks have been assigned in accordance with literature data [48, 49]. Figure 6 also depicts the structures of the lignin sub-units in the samples. In the side chain regions of the HSQC spectra, the β-*O*-4′ aryl ether (A) linkages were identified by the cross peaks at *δ*_C_/*δ*_H_ 74.1/6.03 ppm (*A*_α_) and 88.3/5.46 ppm (*A*_β_), corresponding to the correlation of α-position of *A*_α_-OR and β-position of A-H/*G*, *S*, respectively. Phenylcoumaran (B) constitutes the second most abundant inter-unit (β-5′) formed by coupling monolignol β-position with available 5-position of lignin units. The signals of *δ*_C_/*δ*_H_ 88.3/5.46 ppm (*B*_α_) and 50.5/3.74 ppm (*B*_β_) were ascribed to the correlation of lignin α- and β-position. In addition, the contours at *δ*_C_/*δ*_H_ 85.7/4.73 ppm (*C*_α_) and 54.2/3.05 ppm (*C*_β_) were assigned to resinols (C) showing β-β′ structure. In the aromatic regions in the HSQC spectra, cross-signals from syringyl and guaiacyl lignin units showed prominent correlations at *δ*_C_/*δ*_H_ 103.8/6.58 ppm (*S*_2,6_); 111.1/6.93 ppm (*G*_2_) and 118.7/6.84 ppm (*G*_5,6_), respectively (not shown). Semi-quantitative evaluation of the inter-unit linkages in lignin is classically expressed as the number of inter-unit linkages per 100 aromatic rings (Ar) using the G C_2_-H signals (*G*_2_) as internal standard [50].

As illustrated in the Fig. 6, the HSQC spectra of the pretreated samples showed significant changes in the side chain regions with increased pretreatment severity. The reduced contour intensities of the cross peaks at *δ*_C_/*δ*_H_ 74.1/6.03 ppm (*A*_α_) and 88.3/5.46 ppm (*A*_β_) indeed indicates the degradation of the β-*O*-4′ aryl ether linkages during the pretreatment. Based on the quantified results (Table 5), the content of β-*O*-4′ aryl ether is about 42–45/100 Ar in the untreated LCBs while this content has dramatically decreased in the pretreated residues with increased pretreatment severity. All these data indicate that the depolymerization of the lignin represents the predominant reaction during the pretreatment regardless the type of LCB, and the extent of this process greatly increases with the pretreatment severity [20]. However, the content of β-β′ and β-5′ inter-unit linkages is stable or even slightly increased in the pretreated samples with the exception of miscanthus samples after pretreatment at the most important severity (CSF = 2.8) (Table 5). Surprisingly, the contour intensity of the cross peaks in these miscanthus samples was not detected or did not allow an acceptable quantification. For the other LCB samples, these data coincide nevertheless with recent studies on the structural characterization of grass lignin after steam explosion pretreatment [20, 21, 51]. It could thus be suggested that rearrangement from aryl-ether (β-*O*-4′) linkages to phenylcoumaran (β-5′) and/or resinol (β-β′) occurred during the pretreatment, probably initiated by the homolytic cleavage of β-*O*-4′. Moreover, the previously discussed FTIR data (Fig. 4; Table 3) have shown that some repolymerization and recondensation reactions of the lignin took place in miscanthus and wheat straw during the pretreatment, which are likely two competing reactions during the steam explosion pretreatment. However, the issue of whether the pretreatment leads to condensation of lignin aromatic units still remains partly unexplained even if, as previously suggested, the mechanistic pathway of recondensation could involve the formation of a common carbocation intermediate [52].

**Table 5:** Quantificational results of the lignin by 2D HSQC spectra (results expressed per 100 aromatic rings)

Lignocellulosic biomass	Pretreatment severity (CSF)	β-*O*-4′	β-5′	β-β′	*S*/*G* ratio
*Miscanthus* × *giganteus*	Untreated	42.1	9.9	3.8	1.34
	2.0	13.3	11.7	2.1	0.79
	2.6	8.5	9.1	2.1	0.92
	2.7	4.2	9.1	7.0	0.81
	2.8	ND	ND	ND	ND
Poplar	Untreated	43.1	11.6	6.0	1.29
	2.0	31.0	8.0	9.9	2.23
	2.6	13.0	7.0	9.8	2.09
	2.8	6.8	9.5	12.1	1.95
Wheat straw	Untreated	45.3	8.3	5.6	1.12
	2.0	20.3	11.3	2.1	0.84
	2.6	12.4	10.6	1.9	1.04
	2.7	8.1	9.8	5.0	0.96
	2.8	9.3	9.2	3.9	0.95

*S*/*G* ratio is the ratio *S*_2,6_/*G*_2_

*ND* contour intensities of the corresponding cross peaks not detected or not allowing an acceptable quantification

Besides the β-*O*-4′ aryl ether linkages degradation, the change of the syringyl/guaiacyl (*S*/*G*) ratio was another important structural alteration observed after the steam explosion pretreatment (Table 5). The *S*/*G* ratio was determined on the basis of the number of carbons per aromatic ring in C-2 of guaiacyl units and in C-2,6 of syringyl units [50]. Our data thus suggest that the S-type lignin was more easily degraded in the pretreated miscanthus and wheat straw residues, as revealed by the slightly decreased *S*/*G* ratio (Table 5). This lower *S*/*G* ratio also added some clues to explain the higher content of condensed structures in lignin of pretreated miscanthus and wheat straw residues, because the guaiacyl-type lignin units were found to be more reactive toward condensation. It has been indeed established that acid-catalysed condensation reactions processed most rapidly at the *C*_6_ position [53, 54]. Accordingly, less syringyl units were condensed during the steam explosion compared to the guaiacyl units because of the steric hindrance from the methoxyl groups at the *C*_5_ position. By contrast, an opposite tendency was found for the pretreated poplar residues (Table 5). Here, the *S*/*G* ratio increased, suggesting preferential removal of *G*-units. These results are further supported by FTIR data that did not reveal any significant change of condensed structures in the lignin of the pretreated poplar samples (Table 3).

### Correlating chemical factors to saccharification

**Fig. 7:**
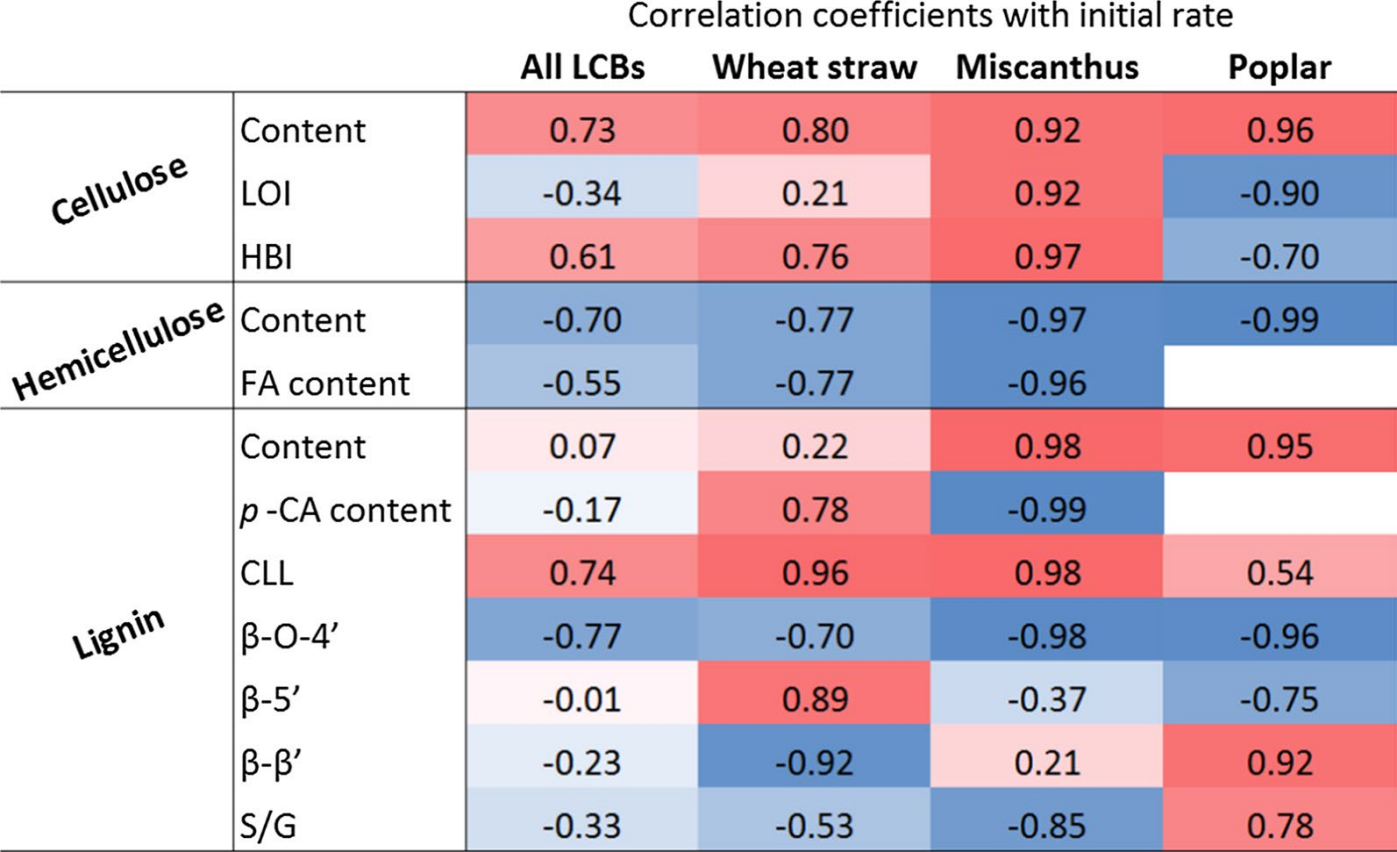
Correlation coefficients between glucose initial rate during saccharification (Table 1) and some polymer-related measured factors. Positive coefficients are in *red*, negative coefficients are in *blue*

With the aim of defining one factor possibly predicting saccharification, simple correlation coefficients were calculated between saccharification initial rate and the factors previously determined and related to cellulose (content, LOI, HBI), hemicellulose (content, FA content) and lignin (content, CLL, *p*-CA content, β-*O*-4′, β-5′ and β-β′) (Fig. 7). First, considering all LCBs, positively correlated factors are cellulose content and CLL while negatively correlated factors are hemicellulose content, FA content and β-*O*-4′ content. More interestingly, considering each LCB separately, different factors are correlated to initial rate conversion. All cellulose-related parameters are highly relevant and positively correlated for miscanthus, while for poplar, LOI and HBI are negatively correlated. In wheat straw, these parameters are not so relevant. Regarding hemicellulose factors, they are very well correlated for miscanthus and poplar, less for wheat straw. For lignin parameters, CLL, which reflects the proportion in condensed structures, shows positive correlations with the initial rate of glucose released. This result agrees well with the negative correlation given by the content in β-*O*-4′ especially in the case of miscanthus. Regarding further lignin structure indicators, the proportion of resinol (β-β′) or phenylcoumaran (β-5′) are relevant parameters for miscanthus and poplar albeit in opposite ways. In addition, relevant correlation is obtained between initial apparent rate of glucose and *S*/*G* ratio. However, grass (wheat straw and miscanthus) and poplar display negative and positive relations, respectively, underlying the effect of the type LCB on the saccharification. Such distinct behaviour can be explained by the presence of phenolic acid, especially PCA which esterifies syringyl units of grass species. At the grass level, PCA is also a relevant factor showing negative and positive relations with the glucose rate for miscanthus and wheat straw, respectively, indicating again some variation in the lignin structure and linkages within the cell wall network. Thus, homolytic cleavage of β-*O*-4′ structures/recoupling reactions are dependent on the lignin monomer composition [20].

Overall, depending on LCB species, different factors should be used to correlate with initial apparent rate of glucose during saccharification. Among them, easiest ones are measured by FT-IR: CLL for wheat straw, HBI and CLL for miscanthus, LOI for poplar, with very good correlation coefficients in the range 0.90–0.98. Notably, these results are dependent on the pretreatment used and inverse correlations have been previously found for other LCBs [55].

## Conclusions

The present study highlights some key structural features impacting the enzymatic saccharification of steam exploded LCBs varying both in pretreatment severity and in biomass type, i.e. *Miscanthus* × *giganteus*, poplar and wheat straw. Altogether, our results confirm that the steam explosion pretreatment represents a versatile pretreatment promoting the glucose release from cellulase-catalysed hydrolysis of woody and non-woody species. The chemical analyses have demonstrated that the pretreatment significantly removes the hemicellulosic fraction, facilitated by the considerable reduction in ferulic acids cross-links, greatly contributing towards the improved cellulose accessibility. Hence, the pretreated residues contain mostly cellulose and lignin, regardless the biomass type. The cellulose structure itself is also altered by the pretreatment, especially through changes in crystallinity. The pretreated poplar indeed showed a less ordered cellulose structure while the opposite pattern was observed after pretreatment of miscanthus and wheat straw samples in spite of similar enzymatic saccharification performances. Thus, it is clear that the cellulose crystallinity cannot be correlated with the higher susceptibility to cellulase-catalysed hydrolysis observed for the pretreated LCBs. Moreover, significant modifications of lignin structure and its redistribution occur, also favouring the cellulose accessibility due to the most exposed cell wall structure as shown by the microscopy images. In light of our results, a possible redistribution mechanism of lignin can originate from: (i) the homolytic cleavage of β-*O*-4′ interlinkages during pretreatment, leading to their depolymerization while (ii) β-β′ and β-5′ linkages are mostly preserved in the residues, regardless the LCB, suggesting that some reorganization/recondensation reactions also took place after pretreatment. Finally, the pretreatment selectively degraded the S-type lignin fragments in miscanthus and wheat straw samples while the pretreated poplar exhibited preferential removal of G-type lignin, showing that the S/G ratio changes cannot explain alone the higher hydrolysis rate in the pretreated residues. Overall, this study provides insights into the fundamental impact of the steam explosion pretreatment on the biomass recalcitrance. Large behaviour differences have been observed between the woody and non-woody biomasses, giving the opportunity to identify some key factors impacting this recalcitrance. Using these parameters should allow to explain and likely to predict the saccharification efficiency depending on biomass type, structure and composition.

## Methods

### Lignocellulosic feedstocks and pretreatment

Both native and steam exploded *Miscanthus* × *giganteus*, poplar and wheat straw residues were provided by the Futurol project (Pomacle, France). During the industrial process, LCB was chopped into smaller pieces (<2 to 3 cm) and used as the raw material. Subsequently, these pieces were presoaked into dilute sulfuric acid (concentrations used are confidential) at room temperature. The presoaked LCB was then transferred to the high-pressure reactor in which the acid-catalysed steam explosion was performed. The operating temperatures and reaction time were also modulated (confidential data). After pretreatment, the treated LCB was recovered and freeze-dried. Moisture content was determined gravimetrically using a halogen moisture analyser (model HR73, Mettler Toledo). Each experimental condition imparted different severity which can be expressed as a combined severity factor (CSF). CSF (log *R*_0_) was calculated using the following equation [56]:

1 $$\log R_{0} = \log_{10} \left( {t*\exp^{{\frac{{\left( {T - T_{\text{R}} } \right)}}{14.75}}} } \right) - {\text{pH}},$$

where *t* is the reaction time (min), *T* is the operating temperature (°C), *T*_R_ is the reference temperature (100 °C), and pH is that of the pretreatment hydrolysate. The different CSF values ranged from 2.0 to 2.8.

### Lignocellulosic samples preparation

All the experiments and measurements described in this work were performed on the same batch of samples. Concerning enzymatic saccharification assays and SEM analyses, both native and steam exploded samples were used as received, without washing or further milling. For other experiments, samples were ball-milled in a 25 mL jar with 20 × 20 mm ZrO_2_ ball bearings using a Retsch MM2000 mixer mill for 2 min to achieve a size below 80 µm. Then samples were washed twice for 2 h in water before filtration and freeze-drying.

### Enzymatic saccharification

The commercial cellulase preparation Cellic CTec2®, courteously provided by Novozymes A/S (Bagsværd, Danemark), was used for enzymatic saccharification assays. Cellulase activity, in terms of “filter paper unit” (FPU), was determined in three repetitions (157.3 FPU/mL) according to the filter paper method using Whatmann no 1 filter paper as standard substrate [57].

Enzymatic saccharification of different substrates (2% w/v) was carried out in 10 mL sodium citrate buffer (0.1 M; pH 5.0) for 48 h at 50 °C and 200 rpm using an enzyme loading of 40 FPU/g-glucan. The reaction mixtures were pre-incubated for 1 h at 50 °C and 200 rpm and then enzymatic hydrolysis was initiated by addition of the cellulase preparation. Control experiments without enzyme were also carried out. Samples were periodically withdrawn and the reaction was stopped by enzyme deactivation for 20 min at 90 °C. Hydrolysates were then sampled for monomeric sugars analysis using high performance anion-exchange chromatography (HPAEC-PAD) using an ICS-5000 + system (Dionex, Sunnyvale, CA, USA) equipped with pulsed amperometric detection and a CarboPac PA1 (2 × 250 mm) column coupled to a CarboPac PA1 (2 × 50 mm) guard column kept at 22 °C. The flow rate was 1.0 mL/min using a gradient method described previously [58]. 2-Deoxy-d-ribose was used as internal standard and quantification was based on calibration curves established using pure standards for arabinose, rhamnose, galactose, glucose, xylose, mannose, galacturonic acid and glucuronic acid.

Apparent initial rates of glucose production were calculated by derivation of a second-order polynomial approximation of the concentration profile, built on experimental data obtained during the first hour of cellulase-catalysed hydrolysis (SigmaPlot 12.0 software, Systat).

### Carbohydrate quantification

Monomeric sugars and uronic acids were released from substrates by a two-step H_2_SO_4_ hydrolysis. Samples (10 mg) were mixed with 125 µL of a 12.0 M H_2_SO_4_ solution for 2 h at room temperature under stirring, followed by dilution to 1.0 M and incubation for 2 h at 100 °C. Hydrolyzed monosaccharides were finally quantified by HPAEC-PAD as described above [58].

### Klason lignin quantification

Milled samples (300 mg) were treated with 3 mL of a 12.0 M H_2_SO_4_ solution at room temperature for 2 h. Distilled water (40 mL) was then added to the slurry to achieve a 2.0 M H_2_SO_4_ final concentration, and the mixture was incubated for 3 h at 100 °C. Solid fraction was then recovered, thoroughly washed and oven dried at 105 °C to a constant weight (weight A). Finally, crucibles containing samples were calcinated in a muffle at 550 °C for 4 h. After cooling to room temperature inside a desiccator, the ash content was gravimetrically determined as well as the acid insoluble lignin content (AIL) by subtraction from A.

Acid soluble lignin was determined by absorbance measurements (280 nm) of the H_2_SO_4_ solution using a UV–Vis spectrophotometer (UV-2401PC, Shimadzu, Kyoto, Japan), and taking into account the interfering absorption of furfural and hydroxymethylfurfural [59].

### Phenolic acids quantification

Samples (40 mg) were added to 10 mL of a 4.0 M NaOH solution and incubated at 170 °C for 2 h for the determination of the total phenolic monomers content while the quantification of the ester-linked monomers was performed using 10 mL of a 2.0 M NaOH solution for 2 h at 35 °C [37, 60]. After cooling to room temperature, pH was adjusted to ~1 with hydrochloric acid and phenolic compounds were extracted three times with diethyl oxide. Ether fractions were pooled and evaporated under reduced pressure (800 mbar). Residues were finally added to 1.5 mL of a H_2_O/methanol solution (1:1 v/v) and quantified using HPLC high-performance liquid chromatography using a Sperisorb S5ODS2 (Waters, RP-18, 250 × 406 mm) column equipped with Waters photodiode array UV detector at 302 nm. A gradient method was used as follows: A (acetonitrile–orthophosphoric acid 15 mM, 10:90 v/v) 100–92% for 6 min; then 92–0% A with solvent B (methanol–orthophosphoric acid 15 mM, 80:20 v/v) 0–50% and C (acetonitrile–orthophosphoric acid 15 mM, 80:20 v/v) 0–50% for 29 min. Internal reference was 3,4,5-trimethoxycinnamic and pure standards of vanillin, vanillic acid, 4-hydroxybenzoic acid, syringic acid, syringaldehyde, *p*-coumaric acid, ferulic acid and sinapic acid (Sigma-Aldrich, St. Louis, MO, USA) were used for the validation of peak compound identification and for the quantification.

### FTIR analysis

FTIR spectroscopic analysis of native and steam exploded LCB was performed using a Thermo Nicolet 6700 FTIR spectrometer. The discs were prepared by mixing 200 mg of spectroscopic grade KBr with 2 mg of milled sample. Spectra were recorded three times in the range 4000–400 cm^-1^ with a resolution of 4 cm^-1^ and 16 scans per sample and corrected for baseline prior to analysis.

### 2D HSQC NMR analysis

DMSO (1.8 mL) and *N*-methylimidazole were added to 100 mg of each ball-milled sample for cell wall dissolution [61]. After acetylation and precipitation into water, samples were centrifuged at 18,600*g* for 10 min (Beckman JLA-10.500 rotor). The pellets were washed twice with water and then centrifuged as previously described [62]. Acetylated sample (~80 mg) was dissolved in 0.6 mL CDCl_3_ in a 5 mm NMR tube. NMR spectra were acquired on a Bruker Biospin Avance III 600 MHz spectrometer using a 5 mm TCI cryoprobe. The ^1^H–^13^C correlation experiment was an adiabatic HSQC experiment (Bruker standard pulse sequence “hsqcedetgpsisp2.2”). HSQC experiments were carried out using the following parameters: non-uniform sampling 50%; 1024 points were acquired in F2 (^1^H) with 64 scans and spectral width of 7211.539 Hz and a relaxation delay of 1 s at 300 K; F1 (^13^C) dimension with spectral width of 33,519.789 Hz was recorded with 386 time increments. Volume integration of contours in HSQC plots used Bruker’s TopSpin 3.1 software. All the spectra were manually phase corrected and calibrated with the CDCl_3_ signal (*δ*_C_ 77.2 ppm, *δ*_H_ 7.26 ppm) used as internal reference. Spectral assignments were characterized in agreement with the literature data [48, 49].

### SEM analysis

Morphology of samples was investigated without sputter coating by scanning electron microscopy using an environmental tabletop electron scanning microscope Hitachi TM-1000 in low-vacuum mode.

### Data and statistical analysis

All the experiments were carried out in triplicate, and the results were expressed as means ± standard deviations. An analysis of variance (ANOVA) was performed on similar results followed by Tukey’s post hoc test for multiple comparisons with a significance level of probability set at *p* < 0.05 (Sigma Plot 12.0 software).

## Abbreviations

2D-HSQC NMR: two-dimensional heteronuclear single-quantum coherence solution nuclear magnetic resonance spectroscopy
AIL: acid insoluble Klason lignin
ASL: acid soluble lignin
CSF: combined severity factor
CLL: cross-linked lignin
FA: ferulic acid
FTIR: Fourier transformed infrared
HBI: hydrogen bond intensity
HPLC: high performance liquid chromatography
HPAEC-PAD: high performance anion-exchange chromatography with pulsed amperometric detection
LCB: lignocellulosic biomass
LCCs: lignin-carbohydrates complexes
LOI: lateral order index
*p*-CA: *p*-coumaric acid
SD: standard deviation
SEM: scanning electron microscopy
